# MOG Antibody‐Associated Disease With Bilateral Deep Gray Matter Involvement in a Child: Unique MRI Findings and Therapeutic Response

**DOI:** 10.1155/crpe/1435159

**Published:** 2026-08-25

**Authors:** Abdulkareem Saymeh, Hamza A. Abdul-Hafez, Alaa Zayed, Maysaa Alawneh, Motee Ashhab, Dima Jabri, Issa Alawneh

**Affiliations:** ^1^ Department of Pediatrics, An-Najah National University, Nablus, State of Palestine, najah.edu; ^2^ Department of Medicine, Faculty of Medicine and Health Sciences, An-Najah National University, Nablus, State of Palestine, duhs.edu.pk; ^3^ Department of Pediatrics, Makassed Hospital and, Al-Quds University, Jerusalem, State of Palestine, alquds.edu; ^4^ Department of Radiology, Rafidia Hospital, Nablus, State of Palestine

**Keywords:** autoimmune encephalitis, demyelination, MOG antibody disease, MOG-IgG antibodies, MOGAD, myelin oligodendrocyte glycoprotein, pediatric neurology, seizures

## Abstract

Myelin oligodendrocyte glycoprotein antibody‐associated disease is an autoimmune inflammatory demyelinating disorder of the central nervous system with diverse clinical and radiological manifestations; however, bilateral symmetric deep gray matter involvement is an exceptionally uncommon presentation in children. We report a previously healthy 3‐year‐old boy who presented with fever, encephalopathy, and focal seizures following a viral illness. Brain magnetic resonance imaging (MRI) demonstrated bilateral symmetric T2/FLAIR hyperintensities involving the basal ganglia, thalami, external capsules, subcortical white matter, and brainstem. Cerebrospinal fluid analysis revealed lymphocytic pleocytosis, and MOG‐IgG antibodies were detected using a cell‐based assay. Extensive infectious, metabolic, mitochondrial, and genetic investigations were unrevealing. Despite treatment with high‐dose intravenous methylprednisolone and intravenous immunoglobulin, the patient showed minimal clinical improvement; however, six sessions of plasmapheresis resulted in marked neurological recovery, with complete clinical and radiological resolution by Day 24. This case expands the recognized neuroimaging spectrum of pediatric MOGAD and highlights the importance of considering MOGAD in children presenting with encephalopathy and atypical bilateral deep gray matter lesions after exclusion of alternative etiologies. It also underscores the potential role of plasmapheresis in severe or steroid‐refractory cases, emphasizing the importance of early diagnosis and prompt immunomodulatory treatment for favorable outcomes.

## 1. Background

Myelin oligodendrocyte glyoprotien (MOG) is a glycoprotein presented widely on the outer surface of the oligodendrocytes and myelin sheath of the nerves. The presence of this protein has a significant role in cell adhesion, stability of oligodendrocytes, and complement response stability [1].

MOG antibody disease (MOGAD) is a recently identified autoimmune inflammatory disease that damages the myelin sheath, showing a wide range of clinical phenotypes including neuromyelitis optica spectrum disorder (NMOSD), optic neuritis (ON), acute disseminated encephalomyelitis (ADEM), myelitis, and multiple sclerosis (MS) [2]. Additionally, it may be associated with anti‐n‐methyl‐d‐aspartate (NMADA) antibody encephalitis, COVID‐19, and teratoma [3, 4]. Currently, after the growing use of cell‐based assay detection methods, MOG‐IgG‐associated encephalomyelitis (MOGEM) has been recognized as immunopathogenetically distinct from NMOSD and other inflammatory demyelinating diseases [5, 6].

Initial diagnosis of MOGEM depends on clinical features, magnetic resonance imaging (MRI) findings, and antibody testing [6]. Previous studies demonstrate that MRI shows abnormal findings in up to 44% of MOGEM patients [1]. However, bilateral symmetric basal ganglia involvement is very rare [7, 8]. To our knowledge, only one pediatric case describing bilateral symmetric basal ganglia and thalamic involvement in MOGAD has previously been reported [7]. Unlike the previously reported case, our patient lacked leptomeningeal enhancement, failed to improve after high‐dose corticosteroids and IVIG, and demonstrated marked clinical and radiological recovery only after plasmapheresis. Therefore, this report expands both the neuroimaging phenotype and the spectrum of therapeutic response in pediatric MOGAD.

## 2. Case Presentation

**FIGURE 1:**
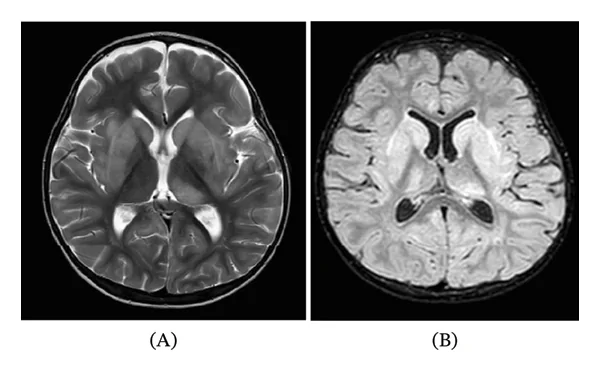
Brain MRI for the patient at the initial presentation: (A) T2 weighted image at the level of the basal ganglia shows bilateral hyperintensity signal involving caudate, putamen, external capsule, and left thalamic. (B) FLAIR axial image at the level of basal ganglia showed the same findings.

A 3‐year‐old male patient, previously healthy with normal antenatal care, developmental milestones, and normal medical history, presented to our hospital with a 2‐day history of focal seizures in the context of recent viral illness, encephalopathy, and hypoactivity. On exam, he was encephalopathic with upper motor neuron signs including hyperreflexia and bilateral Babinski signs. An encephalitis workup was commenced. Initial blood work showed leukocytosis and elevated CRP 73 mg/L. The brain CT scan was unremarkable. CSF analysis showed an elevated cell count (45 cells/mm^3^, predominantly lymphocytes) with normal protein and glucose levels. He was started on ceftriaxone and acyclovir as well as a loading dose of phenytoin and then started on levetiracetam maintenance. Brain MRI demonstrated abnormal high signal intensity on T2/FLAIR images of symmetric involvement of the basal ganglia, thalami, and, to a lesser extent, the external capsules, subcortical cerebral white matter, and the brainstem. The spinal cord and bilateral optic nerves were normal (Figure 1). An electroencephalogram (EEG) revealed findings in keeping with moderate encephalopathy.

**FIGURE 2:**
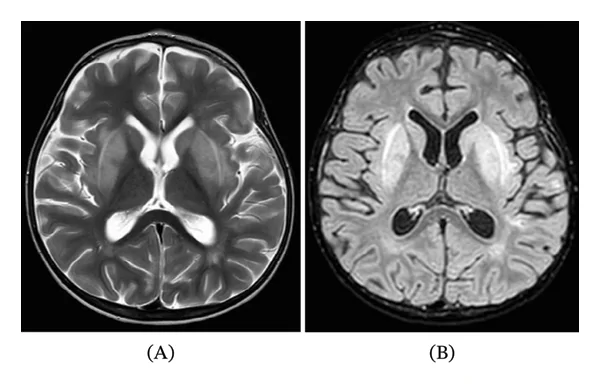
Brain MRI for the patient after 2 weeks from the initial presentation: (A) T2 weighted image at the level of the basal ganglia shows bilateral symmetric hyperintensity signal involving caudate, putamen, and external capsule. (B) FLAIR axial image at the level of basal ganglia showed the same findings.

Autoimmune encephalitis antibodies panel was sent and the patient was started on intravenous methylprednisolone (IVMP) 30 mg/kg for 5 days with no improvement in his encephalopathy and overall clinical status however seizures stopped with no recurrence. Given bilateral symmetric basal ganglia involvement, acute necrotizing encephalopathy (ANEC) was in the differential, and rapid antigen testing for influenza A and B was negative. Moreover, COVID antigen was negative. The metabolic team was consulted given the possibility of biotin‐thiamine basal ganglia‐responsive disease or mitochondrial diseases. A comprehensive infectious workup, including smears and cultures for tuberculosis and other bacteria, as well as viral PCR for herpes simplex virus, enterovirus, and varicella virus, was negative in both the CSF and serum. Due to the absence of improvement despite steroids, intravenous immunoglobulin (IVIG) 2 g/kg was administered over two days. Unfortunately, follow‐up neurological and clinical assessments after IVIG administration showed no significant improvement. The patient underwent a second follow‐up brain MRI which showed striking bilateral symmetric basal ganglia abnormal signal intensity in T2/FLAIR images (Figure 2). Although the clinical presentation and CSF findings align with autoimmune encephalitis, the presence of bilateral symmetric basal ganglia lesions on imaging is very rare to be seen and more in keeping with metabolic or mitochondrial diseases. The patient was started on biotin, thiamin, COQ10, and L Carnitine with no improvement Serum MOG‐IgG was unavailable; CSF testing using a cell‐based assay demonstrated anti‐MOG IgG positivity (titer 1:20). The presence of bilateral symmetric thalami with abnormal T2/FLAIR signal intensity in patients with MOG antibody‐associated encephalomyelitis is extremely rare. Given the diagnostic update, plasmapheresis was commenced. Although current international recommendations favor serum testing because of its higher diagnostic sensitivity, the positive CSF result was interpreted together with the compatible clinical presentation, characteristic MRI findings, exclusion of alternative diagnoses, and therapeutic response. The patient completed six sessions of plasmapheresis over 12 days, after which his clinical and functional status gradually improved over the next two weeks. By Day 24, clinical and radiological follow‐up with MRI demonstrated normal findings, marking a full recovery. Later on, genetic testing for biotin, thiamine basal ganglia response disease, and RANBP2 came back negative. Plasma amino acid and urine organic acids also came back normal. All in keeping with MOG antibody‐associated encephalomyelitis with a unique radiological entity expanding the radiological phenotype of the disease.

## 3. Discussion

MOGAD is an inflammatory CNS disorder marked by immune‐mediated demyelination of the optic nerves, brain, and spinal cord, primarily affecting children [9]. MOGAD has a higher prevalence in males compared to other CNS autoimmune disorders. It often presents as encephalitis or encephalopathy in younger patients, with varying age and gender patterns observed in different regions [10].

MOGAD, characterized by MOG‐IgG antibodies and specific MRI findings, shares similarities with MS and aquaporin‐4 antibody‐positive NMOSD (AQP4‐NMOSD), accounting for up to 40% of AQP4‐negative NMOSD cases [9]. Unlike AQP4‐NMOSD, which primarily affects the optic nerves and spinal cord, MOGAD typically displays broader distributions of T2 hyperintense lesions involving both white and gray matter, particularly affecting the brainstem and deep gray matter structures [9, 11]. While MOGAD can mimic acute disseminated encephalomyelitis (ADEM), it is more likely to relapse, presenting symptoms such as decreased consciousness, generalized seizures, and behavioral changes. Children frequently present with high cerebrospinal fluid cell counts and longitudinally extensive transverse myelitis, whereas adults are more prone to ON [9].

In children, bilateral symmetrical signal intensity in the basal ganglia and thalamus with an encephalitis presentation can arise from various neurological disorders. Hypoxic‐ischemic injury (HIE) typically follows events like cardiac arrest or asphyxia, characterized by T2 hyperintensities and restricted diffusion in the basal ganglia, indicating their high metabolic demand [9]. Metabolic disorders such as Leigh syndrome also show MRI findings of restricted diffusion and T2 hyperintensities in the basal ganglia, periaqueductal gray matter, and brainstem. Biotin‐thiamine‐responsive basal ganglia disease is a distinct clinical disorder characterized by symmetrical basal ganglia involvement [12]. Similarly, ANEC associated with the RANBP2 mutation also presents with bilateral, symmetric basal ganglia involvement [13].

Infections that must be distinguished include Epstein–Barr virus (EBV) encephalitis and Japanese encephalitis. EBV commonly affects the deep gray nuclei, causing T2 hyperintensity in the basal ganglia and thalami, with lesions in the cerebral hemispheres and brainstem [10]. Japanese encephalitis, a mosquito‐borne virus, presents with bilateral symmetrical deep gray matter involvement, and diagnosis relies on exposure history [9]. Given the broad differential diagnosis of bilateral symmetric deep gray matter lesions in children, the distinguishing clinical, laboratory, and imaging features of the major conditions are summarized in Table 1.

**TABLE 1:** Differential diagnosis of bilateral symmetric basal ganglia and thalamic lesions in children presenting with encephalopathy.

Disease	MRI findings	Clinical clues	Distinguishing features
MOGAD	Bilateral gray and white matter lesions; occasional basal ganglia involvement	Encephalopathy, seizures	Positive MOG‐IgG
ADEM	Multifocal asymmetric lesions	Post‐infectious encephalopathy	Usually steroid responsive
Acute necrotizing encephalopathy	Bilateral thalami ± hemorrhage	Viral infection	RANBP2 mutation
Leigh syndrome	Basal ganglia + brainstem	Developmental regression	Elevated lactate
Biotin‐thiamine disease	Symmetric basal ganglia	Episodic encephalopathy	SLC19A3 mutation
Viral encephalitis	Deep gray nuclei depending on virus	Fever, CSF PCR	Positive microbiology

MRI findings in MOGAD often reveal bilateral and asymmetric T2 hyperintense lesions, primarily affecting the brainstem, particularly the pons, with supratentorial white matter involvement in approximately 35.4% of cases. These lesions often extend to deep white matter and deep gray matter structures, such as the thalamus and basal ganglia [14]. While deep gray matter involvement in MOGAD is less frequently observed, it is increasingly recognized as a diagnostic marker, with studies indicating that approximately 29% of patients exhibit these lesions. Spinal MRIs typically show longitudinally extensive T2 hyperintense lesions in both gray and white matter, while optic nerve involvement often presents with bilateral T2 hyperintense signals and potential perioptic nerve sheath enhancement [7, 14]. Leptomeningeal enhancement can indicate inflammation related to infections or autoimmune conditions [15].

In our case, the absence of optic nerve, spinal cord, and leptomeningeal involvement, coupled with predominant bilateral symmetrical gray matter lesions, makes this case atypical in terms of MRI findings. In contrast to a previous report by Shen et al. a year ago, where brain MRI demonstrated symmetrical lesions in the basal ganglia and thalamus alongside leptomeningeal enhancement, this case lacked such enhancement [7]. In this particular previous case, the patient responded well to corticosteroids, with full resolution of symptoms within a week [7] compared to our case which initial treatments with IVMP and IVIG showed limited effectiveness, the implementation of plasmapheresis resulted in significant clinical improvement, with follow‐up MRI demonstrating the resolution of lesions.

Diagnosing (MOG)‐associated bilateral symmetric basal ganglia lesions is crucial due to its rarity and significant clinical implications that result in early and accurate identification of this disease which can guide appropriate immunotherapy promptly ultimately safeguarding neurological function and enhancing the quality of life for affected individuals.

## 4. Conclusion

This case report emphasizes the critical importance of including MOGAD in the differential diagnosis of pediatric encephalitis, especially with bilateral symmetric basal ganglia involvement on MRI. This rare presentation highlights the variability of MOGAD and its potential to mimic other neurological disorders. The significant clinical improvement following plasmapheresis underscores the effectiveness of targeted immunomodulatory therapies in managing severe MOGAD episodes. Accurate and timely diagnosis of MOGAD is essential for selecting appropriate treatments and improving patient outcomes, given its distinct imaging and serological profiles compared to other demyelinating conditions.

## Author Contributions

Abdulkareem Saymeh, Hamza A. Abdul‐Hafez, and Alaa Zayed: drafting and editing the manuscript. Dima Jabri: interpretation of MRI results and preparing the figures. Maysaa Alawneh and Motee Ashhab: verification of clinical data included in the manuscript. Issa Alawneh: critically revised and gave final approval for publication of the paper.

## Funding

The authors have nothing to report.

## Disclosure

All authors have read and approved the manuscript.

## Ethics Statement

The authors have nothing to report.

## Consent

Written informed consent was obtained from the parents of the patient for publication of this case report.

## Conflicts of Interest

The authors declare no conflicts of interest.

## Data Availability

Data sharing is not applicable to this article as no datasets were generated or analyzed during the current study.
